# Structural Plastic Damage Warning and Real-Time Sensing System Based on Cointegration Theory

**DOI:** 10.3390/s24185961

**Published:** 2024-09-13

**Authors:** Qiang Gao, Junzhou Huo, Youfu Wang, Xiaotian Wang, Chongru Wang

**Affiliations:** School of Mechanical Engineering, Dalian University of Technology, Dalian 116024, China; gao13qiang88@163.com (Q.G.); wangyoufu@mail.dlut.edu.cn (Y.W.); wangxiaotian1@126.com (X.W.); wangchongru1@163.com (C.W.)

**Keywords:** cointegration theory, real-time warning, sensing system, warning coefficient, damage alarming

## Abstract

Structural damage can affect the long-term operation of equipment. Real-time damage warning for structures can effectively avoid accidents caused by structural damage. In this paper, a real-time warning method of structural plastic damage based on the cointegration theory is proposed. This method calculates the cointegration relationship between the strain signals at different measuring points, and the stability of the strain signal relationships is also evaluated. The problem of inaccurate detection caused by the error of strain measurement and environmental influence can be eliminated by the comprehensive judgment of strain between asymmetrical measuring points. A real-time damage sensing system is developed in this paper. In order to improve the real-time and practicability of the system, this paper proposes and determines the residual warning coefficient by analyzing the proportion of the strain residuals exceeding the residual threshold. The research on this sensing system has certain value for the engineering application of damage monitoring methods.

## 1. Introduction

The health of structural parts directly affects the service life of equipment [1,2,3]. Under the long-term action of loads, structural parts will suffer a certain degree of damage, which will affect their service life and even cause engineering accidents. Therefore, the accurate monitoring and warning of structural damage can provide a timely reference for equipment maintenance [4,5], which can effectively avoid losses caused by structural damage.

Structural damage monitoring has always been a hot topic in the field of engineering research [6,7]. Common structural damage mainly includes cracks, holes, plastic deformation, structural fractures and other forms. Researchers have conducted relevant research on different types of structural damage using different types of sensors and signal processing methods [8]. Detection methods based on eddy current [9], acoustic emission [10], piezoelectric ultrasonic guided wave [11], electrical impedance [12], visual recognition [13], fiber bragg grating [14] and other technologies each have their respective advantages in the field of structural health monitoring. In recent years, ultrasonic detection technology and machine learning methods have been widely introduced into structural health monitoring [15]. Among them, ultrasonic detection is widely used to monitor cracks in structures [16,17]. Liu et al. studied a wireless sensor based on a nonlinear ultrasonic modulation method to achieve the real-time online monitoring of fatigue cracks [18]. Kim et al. proposed a reference-free ultrasonic guided-wave crack detection method using the polarization characteristics of piezoelectric wafers [19]. With the continuous optimization and development of machine learning methods, they have been applied to monitor crack damages in structures [20,21]. Wang et al. predicted the growth law of fatigue cracks based on the particle filter model [22]. Chen et al. identified cracks in bridge structures based on a deep learning algorithm and realized the integrated application of the algorithm [23]. Zhou et al. used laser ultrasound and the YOLOv5 framework to monitor and identify structural cracks in interference fits [24]. The continuous development of sensor technology has also provided new methods for monitoring crack damage. Xu et al. studied a new layer sensor that monitors the crack extension of steel bridges by different signals at different fatigue stages [25]. Pang et al. studied a sensor based on microstrip antennas that can detect cracks under layers [26]. Strain can accurately reflect changes in structural stress, and the method of identifying damage through strain in a structure has certain advantages. Yu et al. studied full-optical strain FBG with tunable DFB laser demodulation for monitoring tensile and fatigue damage [27]. Pauw et al. used embedded optical fiber sensors to monitor the fatigue damage of structures [28]. Different detection technologies have their own advantages, and the monitoring of structural damage through the fusion of multiple sensors is also being studied by scholars [29]. Qi et al. monitored crack growth during fatigue by integrating strain sensors and piezoelectric guided waves [30]. Wang et al. used a weighted adaptive Kalman filtering-based method to integrate optical fiber and guided-wave information to identify structural fatigue cracks [31]. In summary, most studies on structural damage monitoring focus on monitoring macroscopic damage such as cracks. In actual engineering applications, the plastic deformation of materials often occurs before cracks initiate, and even before obvious cracks appear, the structure has already been destroyed. Therefore, the monitoring and warning of structural damage before crack initiation are of great significance for early maintenance of structures.

Based on the cointegration theory, an efficient and real-time plastic damage warning method is proposed in this paper, which can accurately identify the plastic damage of structures. The method identifies structural plastic damage through the change in the cointegration residual of the strain series between the measuring points. By analyzing the proportion of residuals exceeding the residual threshold, this paper proposes a warning coefficient. And the stability of the real-time alarming of the damage sensing system is improved by comparing the residual and warning coefficient. This method further realizes the application of damage monitoring theory in practical engineering.

## 2. Cointegration Theory

Time series can be divided into two types: unstable and stable time series. Unstable time series refers to a series in which the mean, variance and other statistical data change with time, while stable time series refers to a series in which the statistical data do not change with time. Most of the strain data for structural damage monitoring are unstable series that change with time, and their linear combination may become a series that does not change with time.

For an unstable time series $y_{t}$, after *d* times of difference, it becomes a stationary time series, which is integrated of order *d*, denoted by $y_{t}\sim I\left(d\right)$. For an unstable variable $Y={\left[y_{1},y_{2},\ldots ,y_{n}\right]}^{T}$, its components are all integrated of order 1. If there is a set of linear combination coefficients $\beta =\left[\beta_{1},\beta_{2},\cdots \beta_{n}\right]$, which makes the previous ***Y*** become a stationary series, then $y_{1},y_{2},\ldots ,y_{n}$ satisfies the cointegration relationship, and the equation is shown below.

(1) $$\beta^{T}Y=\beta_{1}y_{1}+\beta_{2}y_{2}+\ldots +\beta_{n}y_{n}\;\sim \;I$$

where $\beta =\left[\beta_{1},\beta_{2},\cdots \beta_{n}\right]$, ***β*^T^*Y*** is residual covariance, and it is a Gaussian noise series with a mean equal to zero.

From the above formula, we can see that the main purpose of cointegration is to seek a long-term stable equilibrium relationship between two sets of unstable time series. If two data series become stable after the first-order difference calculation and are also stable after linear combination, it means that there is a cointegration relationship between them.

An unstable series is more likely to cause pseudo-regression. The significance of the cointegration test is to determine whether the relationship described by its regression equation is pseudo-regression. Therefore, before solving the cointegration residual, it is necessary to test the stationarity of the time series. Commonly used methods include the ADF (Augmented Dickey–Fuller) test and E-G (Engel–Granger) test.

### 2.1. Augmented Dickey–Fuller (ADF) Method

The ADF method is used to test the stability of time series. If there is a time series $y_{t}$, its ADF model is as follows:

(2) $${∆y}_{t}=\rho y_{t-1}+a+\sum_{i=1}^{p-1}b_{i}{∆y}_{i-1}+\varepsilon_{t}$$

where ${∆y}_{i-1}$ is the difference component of $y_{t}$ and *p* and *b_i_* are coefficients. The a represents the constant term. The ρ is the lag order, and $\varepsilon_{t}$ is the residual series. The coefficient ρ is used to determine the stationarity of the time series $y_{t}$. When each series or its difference series is integrated of order 1, the requirements of the cointegration equation are met, and the next step is to continue the E-G test to complete the subsequent solution.

### 2.2. Engel–Granger (E-G) Method

The theoretical basis of the E-G test is to perform a unit root test on the residuals of the regression equation. If the dependent variable can be described by various linear combinations of the independent variables, it means that there is a relatively stable equilibrium relationship between the two. The part that cannot be described forms a residual series. There is no need for serial correlation between the residual series, so the residuals must be stationary. Therefore, the purpose of the E-G method is to verify whether its residual series is stable. The steps of this method are as follows.

Assuming that $x_{t}$ and $y_{t}$ are both integrated of order 1, the residual difference between them is calculated as follows.

(3) $$\varepsilon_{t}=y_{t}-\beta x_{t}-\alpha$$

After the ADF test, if the residual series $\varepsilon_{t}$ is stable, it means that there is a cointegration relationship between the original series $x_{t}$ and $y_{t}$, where α and β are the corresponding cointegration coefficients.

The existence of a cointegration relationship means that the two sets of time series have a stable relationship in the long term, that is, their long-term equilibrium states are interdependent. The cointegration residual is a stable series obtained after eliminating unstable factors. If the cointegration residual changes suddenly, it means that the cointegration relationship has changed, which can be used as a key indicator for damage warning.

## 3. Process of Structural Damage Warning Method

**Figure 1:**
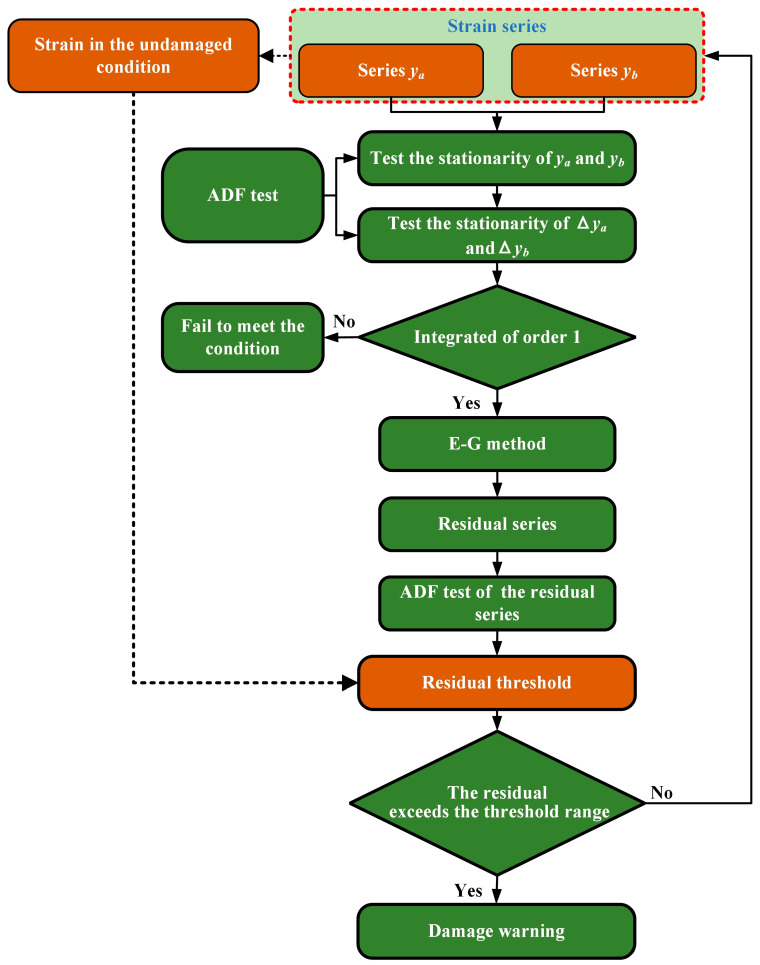
Flowchart of plastic damage warning method based on cointegration theory.

By loading the sample, the strain signals at different measuring points are tested. The residual threshold of damage warning is calculated by the strain signals of the measuring points in the undamaged state. The residual threshold is calculated according to the following method:

(4) $$\left\{\begin{array}{c} RTU=\mu +3\sigma \\ RTL=\mu -3\sigma \end{array}\right.$$

where the RTU is the upper limit of the residual threshold, the RTL is the lower limit of the residual threshold, μ is the residual mean and σ is the residual variance. The flowchart of the damage warning method is shown in Figure 1. First, the stability of the strain data is tested by the ADF method. If the two sets of test strains are unstable, this method is used to evaluate the stability of the corresponding difference series. If the first-order difference of the strain series is stable, the series is integrated of order 1. It meets the requirements of the cointegration equation, and then the E-G test is performed on it. If the E-G result shows that the residual is stable, the residual equation can be constructed. After calculating the residuals of the two series, the ADF stability test is performed on the residual series. If the residuals are unstable, it means that the stability of the two series is destroyed, which also means that structural damage has occurred. If the residuals are stable, the strain series will continue to be calculated to realize the warning of structural damage.

## 4. Test System and Experiment Analysis

### 4.1. Test System and Samples

**Figure 2:**
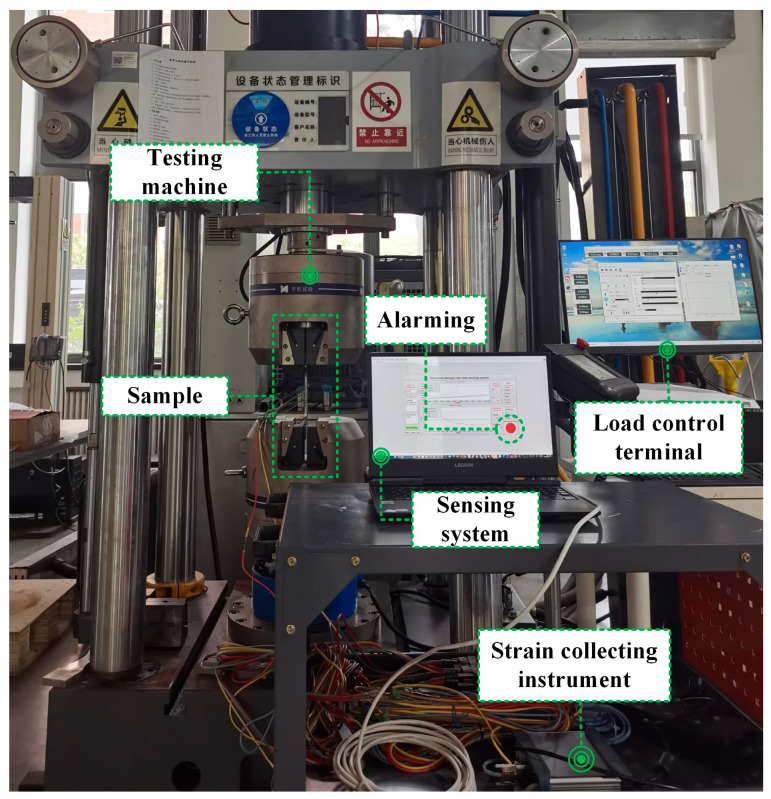
Test system diagram.

As shown in Figure 2, the test system consists of a sample, testing machine, load control terminal, strain collecting instrument and sensing system. The load control terminal controls the testing machine to apply the load to the sample. The strain collecting instrument transmits the collected strain data to the sensing system. The sensor system analyzes the residual of the strain series of multiple channels through the co-arrangement theory and finally realizes the alarm of sample damage.

**Figure 3:**
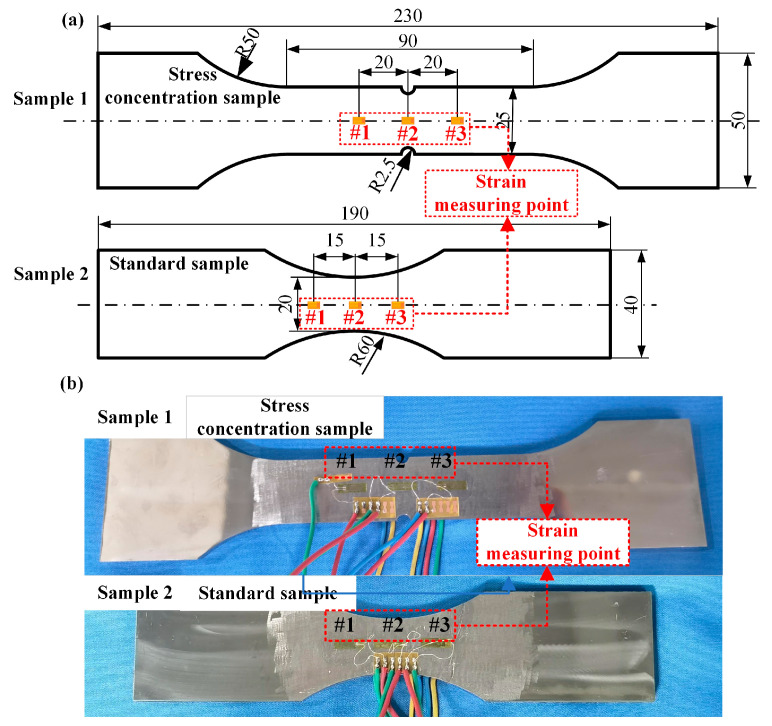
Strain test structure and strain gauge installation position: (**a**) sample structure dimensions and strain gauge installation position; (**b**) sample actual picture (unit: mm).

Figure 3 shows the sample, which is made of Q345D structural steel. Sample 1 is the stress concentration sample, and sample 2 is the standard sample. Strain gauges are pasted in the center and on both sides of samples 1 and 2. The detailed dimensions of the structure and strain gauge pasting are shown in Figure 3a. The thickness of the samples is 5 mm. The actual pictures of the two samples are shown in Figure 3b.

### 4.2. Analysis of Experimental Results

**Figure 4:**
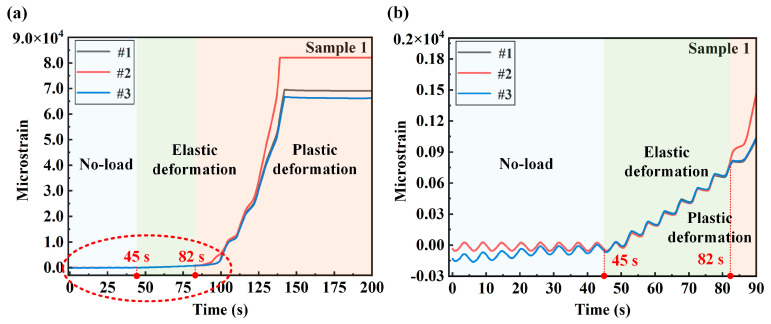
Strain test results of measuring points 1, 2 and 3 of sample 1: (**a**) strain at different measuring points during the entire loading process; (**b**) strain enlarged diagram of the no-loading stage and the plastic deformation stage.

**Figure 5:**
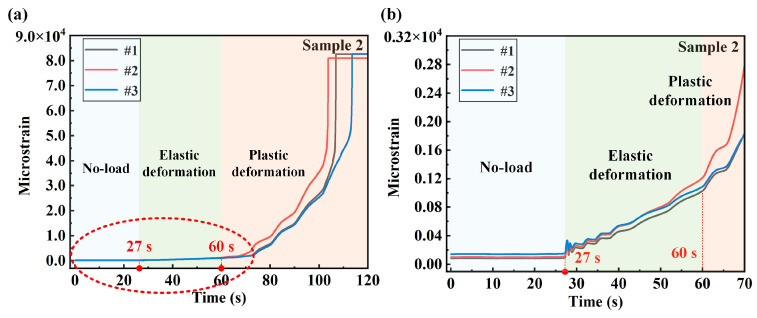
Strain test results of measuring points 1, 2 and 3 of sample 2: (**a**) strain at different measuring points during the entire loading process; (**b**) strain enlarged diagram of the no-loading stage and the plastic deformation stage.

By applying tensile force to the sample, the strain signal of the sample measuring point is tested. Each strain channel is synchronously sampled with a sampling rate of 200 Hz. The strain test results of different measuring points of the two samples are shown in Figure 4 and Figure 5, respectively. The signal of the entire test process is divided into three stages: the no-load stage, the elastic deformation stage and the plastic deformation stage. As shown in Figure 4a, 0–45 s is the no-load stage, and the strain signal tested at this time is the noise of the system. Next, 45–82 s is the elastic deformation stage. At 45 s, the test machine starts to load the sample with a loading rate of 0.5 kN/s. The enlarged curve of the no-load stage and the elastic deformation stage is shown in Figure 4b. It can be seen from the figure that the strain signals of each measuring point from 45 to 82 s show a linear growth trend. It can be judged by Hooke’s law that this stage belongs to the elastic deformation stage. After 82 s of continuous loading, the sample slowly enters the plastic deformation stage. It can be seen from Figure 4a that the strain of each measuring point increases rapidly after entering the plastic zone. The enlarged view of Figure 4b shows that the strain growth rate of measuring point 2 located at the stress concentration position is greater than the strain growth rate of the symmetrically arranged measuring points 1 and 3.

The strain changes of sample 2 during the entire loading process are also analyzed based on the strain test data in Figure 5, and the loading parameters are consistent with those of sample 1. In Figure 5a, the test machine starts loading at 27 s, the strain and load of the sample are linearly related before 60 s and the sample is in the elastic deformation area. It can be clearly seen from Figure 5a,b that after 60 s, with the continuous loading of the load, the strains of the three measuring points of sample 2 gradually change and increase. After 60 s, the sample begins to slowly undergo plastic deformation, and the strain also increases rapidly until the strain gauge fails and the structure is damaged. Similarly, the strain growth rate of measuring point 2 at the center position is greater than the strain growth rate of symmetrical points 1 and 3 when entering the plastic deformation stage. For engineering structural parts, they can work normally in the elastic deformation stage and can still return to the initial state after unloading. Once the structure undergoes plastic deformation, it will cause irreversible damage to the structure and cannot return to the initial state after unloading. Irreversible structural damage will occur after the structural part enters the plastic stage, so accurate monitoring of it can provide timely warning of structural damage.

**Figure 6:**
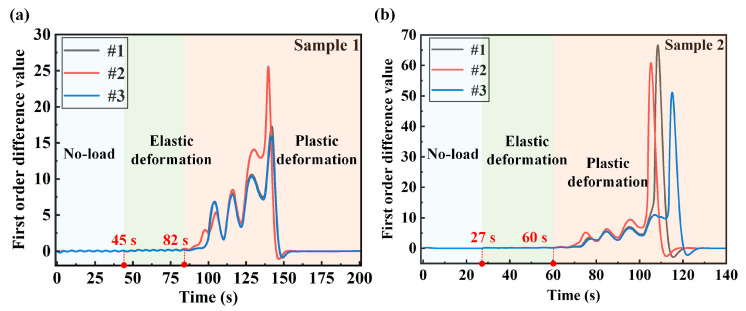
First-order difference series of strain at measuring points 1, 2 and 3: (**a**) strain difference series of sample 1; (**b**) strain difference series of sample 2.

According to the cointegration theory, the strain series needs to be integrated of order 1 in order to conduct a stability assessment. The strain and force changes of the structural parts are linear in elastic deformation. Figure 6 is a first-order difference series of the strain signal at each measuring point. Sample 1 is in the no-load and elastic deformation stages from 0–82 s, and the first-order difference series of the two stages shows the same law. The strain first-order difference series of sample 2 also maintains the same law of the first-order difference in the no-load and elastic deformation stages from 0 to 60 s. After calculating the difference series, the stability of the difference series needs to be checked.

The strain signals of the first 25 s of sample 1 and sample 2 are selected as the stability test data. The ADF test is performed on each strain series and the first-order difference series of the strain, respectively, and the verification results are shown in Table 1 and Table 2. Among them, *y*_1_, *y*_2_ and *y*_3_ are the strain series corresponding to different measuring points of the sample, and Δ*y*_1_, Δ*y*_2_ and Δ*y*_3_ are the first-order difference series of the corresponding strains. By comparing the ADF check value with the ADF critical value at the 1% and 5% significance levels, if the ADF check value of the series is less than the critical value, it means that the series is stable. If the ADF check value of the series is greater than the critical value, the series is unstable. It can be seen from the ADF test results in Table 1 and Table 2 that the ADF test values of series *y*_1_, *y*_2_ and *y*_3_ are all greater than the critical values of 1% and 5%, so the strain series are all unstable. The first-order difference series Δ*y*_1_, Δ*y*_2_ and Δ*y*_3_ are all stable after verification. The strain series of sample 1 and sample 2 are both unstable after verification, and their first-order series are stable. Therefore, the strain series of the two samples are all integrated of order 1, which meets the cointegration requirements.

The threshold of the residual is calculated using the selected data. The cointegration equation of the strain series between different measuring points is constructed by the E-G method, and then the residual is calculated by the cointegration equation. The parameters of the cointegration equation of the strain series of each measuring point are shown in Table 3 and Table 4. Strain series #1–#2 represents the combination of data from measuring points 1 and 2. The thresholds of the strain residual of sample 1 and sample 2 are calculated by Formula (4), and its parameters are shown in Table 5 and Table 6. In addition, the threshold mean value calculated by the three groups of data is calculated in the table, and it is used to judge whether the limit is exceeded.

The ADF method is used to test the stability of the first-order different residual series. From the test results of Table 7 and Table 8, by comparing with the 1% and 5% critical values, the ADF test results of the residuals between each series are all less than the critical value, satisfying the stable condition. And it can be seen that the cointegration residuals between different strain series are all stable. Therefore, there is a cointegration relationship between the strains of the three measurement points of sample 1 and sample 2.

**Figure 7:**
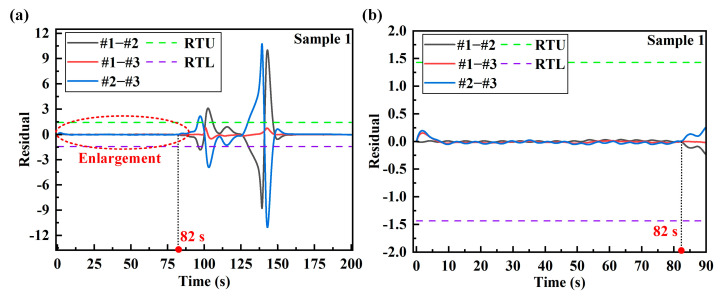
Calculation results of residual at the measuring point of sample 1: (**a**) calculation results of residual during the whole process; (**b**) enlarged residual during the no-load stage and the elastic deformation stage.

**Figure 8:**
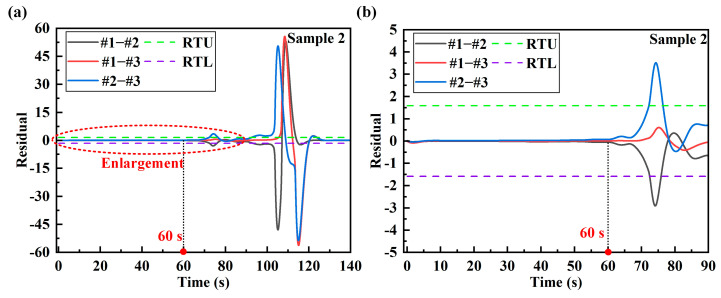
Calculation results of residual at the measuring point of sample 2: (**a**) calculation results of residual during the whole process; (**b**) amplified residual during the unloaded stage and the elastic deformation stage.

The corresponding residual series is calculated for the strains of the three stages. By comparing the residual calculation results with the residual threshold, the warning of plastic deformation damage is realized. Figure 7 and Figure 8 are the residual calculation results of the strain series between the measuring points of sample 1 and sample 2, respectively. For sample 1, the stage of 0–82 s corresponds to the no-load and elastic deformation stages, and its curve is enlarged, as shown in Figure 7b. It can be seen from the enlarged residual diagram that the residual calculation results of the two groups #1–#2 and #2–#3 do not exceed the RTU and RTL before the plastic deformation stage, showing a stable state. With the continuous loading of force, the structural parts began to slowly enter the plastic deformation stage after 82 s. At this time, the relationship between strain and force at each measuring point becomes a nonlinear relationship. Since measuring points 1 and 3 are arranged on the symmetrical side of the stress concentration part of sample 1, the cointegration residual of #1–#3 changes little after plastic deformation. The residual verification results show that the strain cointegration relationship between the stress concentration measuring point and the symmetrical measuring point is very sensitive to plastic deformation. After plastic deformation occurs, the residual value will deviate from the original stable state. As the degree of plastic deformation increases, the residual value gradually increases. The change in the structural damage state can be clearly seen through the change in the residual value.

The residual calculation results of sample 2 are analyzed. As shown in Figure 8, before 60 s of loading, the residual results calculated for the sample in the no-load and elastic deformation stages do not exceed the RTU and RTL. As shown in Figure 8b, after entering the plastic deformation stage, the residual calculation results increased significantly, and the residual stability is destroyed at this time. After the material enters the plastic deformation, the strain relationship changes at the center measuring point and the symmetrical measuring point are very obvious. However, for the symmetrically arranged #1–#3 combination, the residual changes are small just after entering the plastic deformation. In the later stage of plastic deformation, the residual changes significantly. The structural damage warning through residual values needs to be calculated under the action of dynamic loading. After 160 s and 130 s in Figure 7 and Figure 8, the loading stops, the strains are all stable values of the mean and the residual cannot be judged at this time.

Accurate warning of structural damage through the cointegration method requires calculation and analysis based on the relatively stable relationship between the strains of the two measuring points. For the measuring points of the structural parts, the strains of the asymmetric points of the structure should be selected to evaluate the stability of the cointegration relationship. Within the elastic deformation range of the structure, the strains of the two points are linearly related, and their residuals show a certain stability. After the structure enters the plastic deformation, the strains of the asymmetric measuring points will obviously destroy their original stable relationship, and then the warning of structural damage is performed by comparison with the residual threshold.

## 5. Real-Time Damage Sensing System

**Figure 9:**
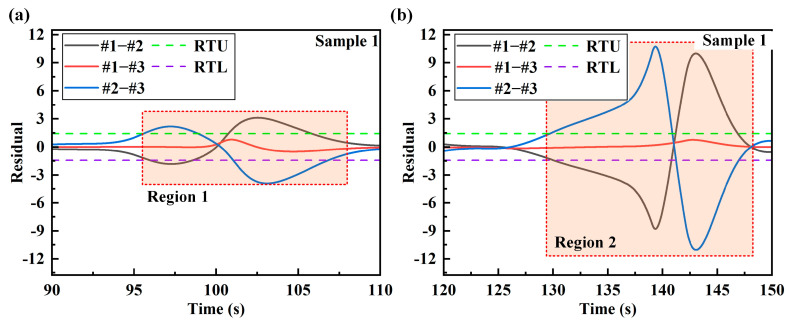
The regions of sample 1 in which the residual exceeds the residual thresholds: (**a**) the labeled figure of region 1; (**b**) the labeled figure of region 2.

**Figure 10:**
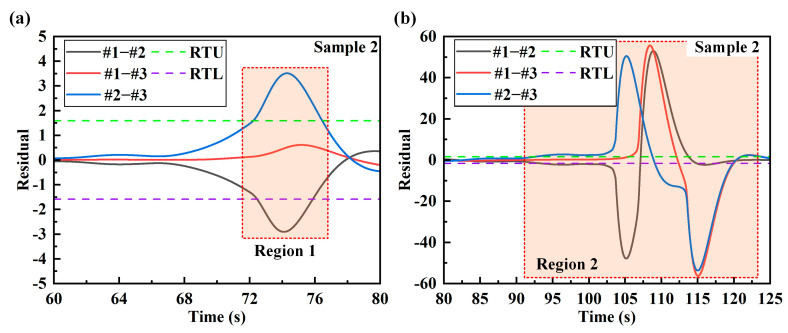
The regions of sample 2 in which the residual exceeds the residual thresholds: (**a**) the labeled figure of region 1; (**b**) the labeled figure of region 2.

In order to ensure the stability of the system alarm, the warning coefficient *q* is proposed in this paper. The warning coefficient is obtained by analyzing the data. In addition, the warning coefficient should be determined according to the sampling data and sampling frequency. The warning coefficient is the upper warning limit of the proportion of data exceeding the residual threshold in the sampled data. A too-large warning coefficient will lead to no warning after damage occurs in the structure. A too-small warning coefficient may be disturbed by unstable factors such as noise in the actual load, resulting in a false alarm. In Figure 9 and Figure 10, the regions in which the residuals exceed the residual thresholds are marked. In 3000 data samples, the proportion of residuals that exceed the threshold is calculated, as shown in Table 9. According to the data analysis in the table, the smallest proportion is 0.19. In practical applications, the residual threshold setting of the damage sensing system needs to be less than 0.19. Since the sampling data of the real-time sensing system is phased sampling, in order to improve the stability of the system alarm, the warning coefficient of the two samples is set to 0.05.

**Figure 11:**
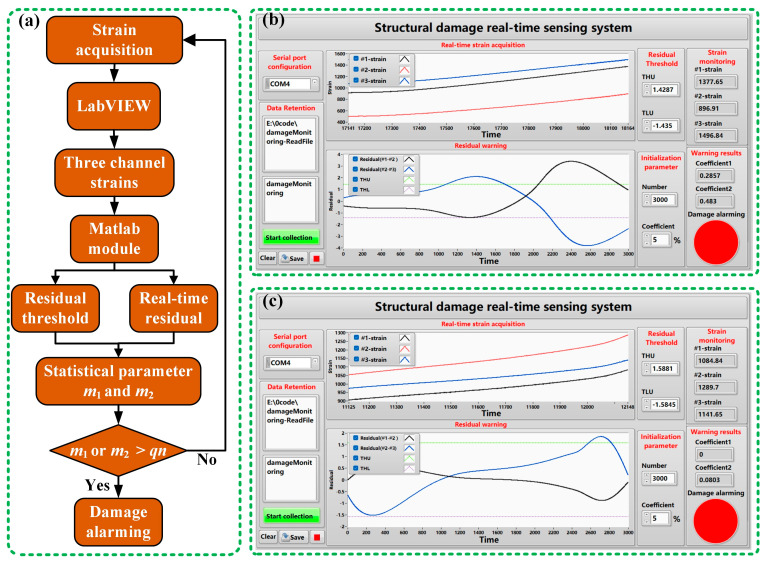
Structural damage sensing system: (**a**) system workflow diagram; (**b**) damage warning result of sample 1; (**c**) damage warning result of sample 2.

The warning of structural damage is inseparable from the real-time calculation and interactive display of a sensing system. In this paper, an intelligent damage sensing system is developed by combining the hybrid programming of LabVIEW 2015 and MATLAB R2023b. The workflow of the sensing system is shown in Figure 11a. The strain-collecting instrument collects the strain data of the sample in real time and transmits the data to the sensing system. And the sensing system packages the strain data of three measuring points into *n* sampling data and transmits them to the MATLAB module. According to the analysis conclusion of this paper, the strain series of measuring points 1 and 2 (#1–#2) and measuring points 2 and 3 (#2–#3) are combined. In order to improve the stability of the alarming function of the sensing system, the system counts the number of *m*_1_ (#1–#2) and *m*_2_ (#2–#3) of the calculated residuals exceeding the threshold in *n* sampling data in real time. The accuracy of the residual calculated for small sampling data will decrease, and too much sampling data will affect the real-time alarming of the sensing system. Through testing and analysis, the *n* of the sensing system is 3000. The MATLAB module calculates the corresponding residuals based on the two sets of data sampled from #1–#2 and #2–#3. Then, the proportion of the two sets of residuals in *n* data is counted in LabVIEW. If the proportion of any set exceeding the threshold is greater than *q*, the result will be reported to the sensor system for damage alarming. Through the continuous sampling and analysis of the sensing system, the warning of structural damage can be achieved in real time. Figure 11b,c are the interface displays of the system damage warning for sample 1 and sample 2, respectively.

**Figure 12:**
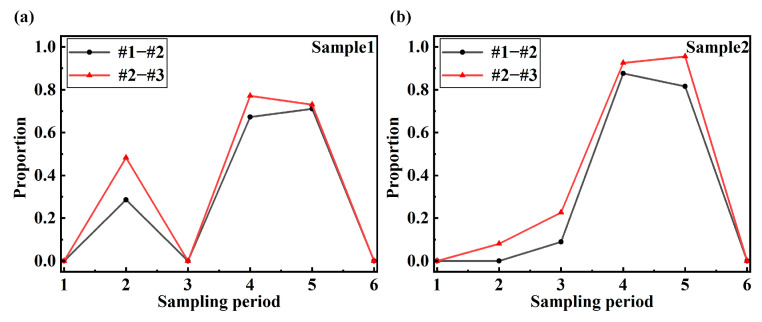
Warning coefficient of structural damage sensing system: (**a**) warning coefficient of sample 1; (**b**) warning coefficient of sample 2.

Figure 12 shows the monitoring value of the warning coefficient of the structural damage sensing system. In the no-load and elastic deformation stages, the damage warning coefficient is 0, and the system does not issue an alarm. In Figure 12, all 3000 data samples correspond to a sampling stage. From the beginning of the damage to structural destruction, the damage warning coefficient basically increases gradually. Once the structure is destroyed, the stress change in the structure is a constant value, and the damage warning coefficient becomes 0. It is worth noting that in the third warning stage of sample 1, both damage coefficients become 0, indicating that after the damage occurs in the first stage, the strain in the structure produces a stable fluctuation, causing the residual to return to a stable state. After continuous loading, the warning coefficient of the structure is greater than the warning coefficient of the second stage, and the warning of damage can still be achieved. It can be seen from the curve of the warning coefficient that under the condition of a sampling number of 3000, a sampling frequency of 200 Hz and a warning coefficient of 0.05, the damage warning accuracy of sample 1 is 75%, and the damage warning accuracy of sample 2 is 100%. Therefore, different structural forms will have a certain impact on the accuracy of the warning system. A comparison of existing damage monitoring studies is presented in Table 10.

## 6. Conclusions

Based on the cointegration theory, this paper proposes a structural damage warning method. This method realizes the warning of structural damage by calculating the cointegration residual between two measuring points in the structure, avoiding the uncertainty of the warning of the single strain threshold method. The strain cointegration residuals of stress-concentrated samples and standard samples are analyzed. The residual analysis results show that the residual of the strain series between two asymmetric measuring points is more sensitive to the plastic deformation damage of the structure. The calculated residual is compared with the threshold value in the residual steady state to realize the identification of structural damage. In order to improve the engineering practicality of the method, this paper combines LabVIEW and MATLAB to develop a real-time structural damage sensing system. The system calculates the residual warning coefficients of the two groups of strain series #1–#2 and #2–#3 in real time and comprehensively considers the two prediction results to warn of structural damage. The test results show that under the conditions of a sampling number of 3000, a sampling frequency of 200 Hz and a warning coefficient of 0.05, the system alarm accuracy for the stress concentration sample is 75%, and for the standard sample it is 100%. Due to different structural characteristics, in actual applications, the system prediction accuracy for different types of structures will vary. The proposed method further realizes the engineering application of structural damage monitoring.

## Figures and Tables

**Table 1:** ADF check results of strain series and first-order difference series of sample 1.

Series	ADF Test Value	1% Critical Value	5% Critical Value	Stability
*y*_1_	−1.0398	−2.5690	−1.9416	Unstable
∆*y*_1_	−99.1729	−2.5690	−1.9416	Stable
*y*_2_	−1.0791	−2.5690	−1.9416	Unstable
∆*y*_2_	−101.1411	−2.5690	−1.9416	Stable
*y*_3_	0.8419	−2.5690	−1.9416	Unstable
∆*y*_3_	−95.9264	−2.5690	−1.9416	Stable

**Table 2:** ADF check results of strain series and first-order difference series of sample 2.

Series	ADF Test Value	1% Critical Value	5% Critical Value	Stability
*y*_1_	−0.1069	−2.5690	−1.9416	Unstable
∆*y*_1_	−121.2684	−2.5690	−1.9416	Stable
*y*_2_	−0.2294	−2.5690	−1.9416	Unstable
∆*y*_2_	−123.6009	−2.5690	−1.9416	Stable
*y*_3_	−1.1644	−2.5690	−1.9416	Unstable
∆*y*_3_	−111.5629	−2.5690	−1.9416	Stable

**Table 3:** Cointegration equation parameters for different strain series of sample 1.

Strain Series	β	α
#1–#2	1.0217	1.7590
#1–#3	0.6183	45.1140
#2–#3	0.6055	42.4706

**Table 4:** Cointegration equation parameters for different strain series of sample 2.

Strain Series	β	α
#1–#2	1.5264	22.3674
#1–#3	−0.1026	−126.8029
#2–#3	−0.0400	−95.5583

**Table 5:** Residual mean, variance and upper and lower limits of different strain series of sample 1.

Strain Series	*μ*	*σ*	RTU	RTL
#1–#2	−1.3747 × 10^−4^	0.4373	1.3118	−1.3121
#1–#3	−0.0048	0.5002	1.4958	−1.5054
#2–#3	−0.0046	0.4943	1.4785	−1.4876
Mean value	N/A	N/A	1.4287	−1.4350

**Table 6:** Residual mean, variance and upper and lower limits of different strain series of sample 2.

Strain Series	*μ*	*σ*	RTU	RTL
#1–#2	−7.7404 × 10^−4^	0.7447	2.2332	−2.2348
#1–#3	5.5210 × 10^−4^	0.4254	1.2769	−1.2658
#2–#3	6.5017 × 10^−4^	0.4179	1.2543	−1.2530
Mean value	N/A	N/A	1.5881	−1.5845

**Table 7:** ADF test results of strain residual series of sample 1.

Strain Series	ADF Test Value	1% Critical Value	5% Critical Value	Stability
#1–#2	−71.4768	−2.5821	−1.9416	Stable
#1–#3	−119.5111	−2.5821	−1.9416	Stable
#2–#3	−120.6987	−2.5821	−1.9416	Stable

**Table 8:** ADF test results of strain residual series of sample 2.

Strain Series	ADF Test Value	1% Critical Value	5% Critical Value	Stability
#1–#2	−106.9975	−2.5821	−1.9416	Stable
#1–#3	−119.1516	−2.5821	−1.9416	Stable
#2–#3	−116.0702	−2.5821	−1.9416	Stable

**Table 9:** The number and proportion of residuals exceeding the threshold in different regions.

Samples	Regions	#1–#2	Proportion	#1–#3	Proportion	#2–#3	Proportion
Sample 1	Region 1	570	0.19	0	0	652	0.22
		977	0.33	0	0	1141	0.38
	Region 2	1086	0.36	0	0	2253	0.75
		2172	0.72	0	0	1113	0.37
Sample 2	Region 1	0	0	0	0	814	0.27
		709	0.24	0	0	0	0
	Region 2	2857	0.95	1304	0.43	3276	1.09
		1518	0.51	1649	0.55	2357	0.79

**Table 10:** Comparison of studies on damage monitoring.

Studies	Sensor	Monitoring Damage Type	Monitoring System	Real Time
Qian et al. [32]	Strain sensor	Crack	No	No
Kontsos et al. [33]	Piezoelectric sensor	Crack initiation	No	No
Xiang et al. [34]	Piezoelectric sensor	Fatigue crack	No	No
Chen et al. [35]	Eddy current sensor	Fatigue crack	No	Yes
Yuan et al. [36]	Piezoelectric sensor	Impact	Yes	Yes
This paper	Strain sensor	Early plastic damage	Yes	Yes

## Data Availability

Data are contained within the article.
